# Role of Areca Nut Induced TGF-β and Epithelial-Mesenchymal Interaction in the Pathogenesis of Oral Submucous Fibrosis

**DOI:** 10.1371/journal.pone.0129252

**Published:** 2015-06-24

**Authors:** Ila Pant, Neeraj Kumar, Imran Khan, Somanahalli Girish Rao, Paturu Kondaiah

**Affiliations:** 1 Department of Molecular Reproduction, Development and Genetics, Indian Institute of Science, Bangalore, Karnataka, India; 2 Department of Oral and Maxillofacial Surgery, D.A. Pandu Memorial—R.V. Dental College and Hospital, Bangalore, Karnataka, India; Boston University Goldman School of Dental Medicine, UNITED STATES

## Abstract

Areca nut consumption has been implicated in the progression of Oral Submucous fibrosis (OSF); an inflammatory precancerous fibrotic condition. Our previous studies have demonstrated the activation of TGF-β signaling in epithelial cells by areca nut components and also propose a role for epithelial expressed TGF-β in the pathogenesis of OSF. Although the importance of epithelial cells in the manifestation of OSF has been proposed, the actual effectors are fibroblast cells. However, the role of areca nut and TGF-β in the context of fibroblast response has not been elucidated. Therefore, to understand their role in the context of fibroblast response in OSF pathogenesis, human gingival fibroblasts (hGF) were treated with areca nut and/or TGF-β followed by transcriptome profiling. The gene expression profile obtained was compared with the previously published transcriptome profiles of OSF tissues and areca nut treated epithelial cells. The analysis revealed regulation of 4666 and 1214 genes by areca nut and TGF-β treatment respectively. The expression of 413 genes in hGF cells was potentiated by areca nut and TGF-β together. Further, the differentially expressed genes of OSF tissues compared to normal tissues overlapped significantly with areca nut and TGF-β induced genes in epithelial and hGF cells. Several positively enriched pathways were found to be common between OSF tissues and areca nut +TGF-β treated hGF cells. In concordance, areca nut along with TGF-β enhanced fibroblast activation as demonstrated by potentiation of αSMA, γSMA and collagen gel contraction by hGF cells. Furthermore, TGF-β secreted by areca nut treated epithelial cells influenced fibroblast activation and other genes implicated in fibrosis. These data establish a role for areca nut influenced epithelial cells in OSF progression by activation of fibroblasts and emphasizes the importance of epithelial-mesenchymal interaction in OSF.

## Introduction

Oral submucous fibrosis is prevalent in South and South East Asia [1]. It is a pre-cancerous condition characterized by inflammation, epithelial atrophy and trismus of the oral cavity due to excessive extracellular matrix deposition [2,3]. Extracellular matrix remodeling including deregulation of synthesis and degradation of collagen, up regulation of pro-fibrotic Transforming growth factor-β (TGF-β) and down regulation of Bone Morphogenic Protein 7 (BMP7) are characteristic features of OSF [4,5,6]. Habit of areca nut chewing is considered as the most probable etiological factor in OSF manifestation [7,8], which is supported by a mouse model [9]. The alkaloid and polyphenol components of areca nut were found to induce and activate TGF-β in epithelial cells [10]. Earlier studies documented increased collagen content in OSF derived fibroblasts [11] and arecoline treated mucosal fibroblasts [12]. A recent report highlights activation of mucosal fibroblasts by areca nut extract suggesting involvement of the PLC/IP3/Ca2+/Calmodulin and Rho signaling pathways along with actin filament polymerization [13]. However, the response of fibroblasts to areca nut together with TGF-β representing OSF pathology is not well studied. Therefore, to gain further insights we studied the effects of areca nut with or without TGF-β on human gingival fibroblasts by transcriptome profiling. The profile obtained was further compared with the transcriptome of OSF tissues and areca nut induced transcriptome in epithelial cells [6,10]. These data demonstrate the involvement of both areca nut and epithelium derived TGF-β in altering fibroblast phenotype, highlighting the importance of epithelial mesenchymal interaction in OSF.

## Materials and Methods

This study has been approved by the Institutional Ethics Committee of DA Pandu Memorial RV Dental College and Hospital. Informed written consent of the participants has been obtained.

The study is designed to understand the role of areca nut on the modulation of fibroblasts that is essential in the manifestation of oral submucous fibrosis. This has been accomplished by treating the human gingival fibroblasts (hGF) with areca nut extract (with or without TGF-β) and subsequent transcriptome profiling and qPCR. The expression profiles were compared to the transcriptome profile of OSF tissues to arrive at possible genes/pathways that may be essential to drive OSF progression. Details of the protocols are as follows:

### Areca nut extract preparation

Previously described protocols were followed for areca nut water extract preparation [10,14,15]. Dried and de-husked areca nut was ground to powder and extracted using constant stirring in 100 ml de-ionized water at 4°C for 4 hours. This was filtered through a sintered glass funnel followed by lyophilization. The lyophilized form was re-dissolved in de- ionized water. The extract was filtered through 0.2 micron filter, lyophilized again and stored at 4°C. The powder obtained was weighed and dissolved in filtered de-ionized water for treatment purposes and was stored at -20°C. To avoid repeated freeze thaw cycles once dissolved; extract was stored in aliquots.

### Cell lines and treatments

Primary human gingival fibroblast cells (hGF) [16] and HaCaT cells [17] were cultured as described [10]_._ For the microarray experiments and validations; hGF cells were serum deprived for 24 hours followed by treatment with sub-cytotoxic dose of 5 μg/ml areca nut water extract with or without 5 ng/ml of TGF-β (R&D systems, USA) for 72 hours. To study the epithelial mesenchymal interaction, conditioned media from HaCaT cells was collected as follows. Confluent cultures of HaCaT cells were serum starved for 24 hours followed by 10 μM ALK5 inhibitor (TGFβRI inhibitor, SB 431542, Sigma-Aldrich, USA) [18] treatment 2 hours prior to areca nut treatment (5 μg/ml). Meanwhile hGF cells were serum deprived for 24 hours such that the treatment time point coincided with completion of 48 hour treatment on HaCaT cells. At this time point, the condition medium of areca nut (with or without ALK5 inhibitor; SB 431542) treated HaCaT cells was transferred to hGF cells and simultaneously direct treatment of areca nut with or without ALK5 inhibitor; SB 431542 was also performed and both were maintained for 48 hours.

### Tissue Samples, RNA isolation and real time RT-PCR

Tissue samples of OSF and normal subjects were procured from D.A Pandu Memorial R.V Dental College and Hospital, Bangalore. This study has been approved by the Institutional Ethics Committee of DA Pandu Memorial RV Dental College and Hospital. Informed written consent of the participants has been obtained. Normal oral tissues were obtained from non-OSF patients who underwent third molar extraction. All the tissues were evaluated by a pathologist.

Total RNA was isolated from homogenates of tissues, hGF and HaCaT cells using TRI reagent (Sigma-Aldrich, St. Louis, USA) as per manufacturer’s protocol. For RT-PCR, 2 micrograms of RNA was converted to cDNA using high capacity cDNA synthesis kit (Applied Biosystems, Foster City, USA). Semi-quantitative PCR was performed using DreamTaq Green PCR 2X master mix (Thermo Scientific). 20 ng of cDNA per 20 μL reaction containing gene specific expression primers was used. Real time PCR was performed using ABI Prism 7900HT sequence detection system. 20 ng of cDNA per 10 μl reaction was used for real time PCR analysis using Dynamo SYBERgreen 2X mix (Finnzymes, Finland) along with the specific primer pair.[10]. The sequences of the primers used are enlisted in Table 1. RPL35A expression was used for normalization [19]. Differential expression of genes was determined using the formula

δCt=Ctgene−CtRPL35A

δδCt=δCttreated−δCtuntreated

$$Fold\;Change\;(FC)=2^{-\delta \delta \;Ct}$$

**Table 1:** List of primers used for PCR.

S. No.	GENE	FORWARD PRIMER 5’-3’ sequence	REVERSE PRIMER 5’-3’ sequence	DETAILS
**1**	αSMA	CAGCCAAGCACTGTCAGG	CAATGGATGGGAAAACAGC	150 bp, 59.5°C
**2**	γSMA	CCTCAGTCACTGGGAGAAGAA	ATCATCTCCTGCGAAGCCT	150 bp, 59.5°C
**3**	BMP1	ACAGCACAGGCAACTTCTCC	GGGACGTGAAGTTCAGGATG	117 bp, 59.5°C
**4**	CD248	TGCCAACGTGTGTCTTTTTG	GTTCTGTTGGGCTCTTGCTC	141 bp, 59.5°C
**5**	COL15A1	CAGTGCTGGTGTCTGCTGAT	GACAAAGGATACGGACGAGG	150 bp, 59.5°C
**6**	CTGF	CAGCATGGACGTTCGTCTG	CCAACCACGGTTTGGTCCTT	117 bp, 59.5°C
**7**	EDN1	CGAGCACGTTGTTCCGTATG	CAGCCCTGAGTTCTTTTCCTG	164 bp,55°C
**8**	EGR2	GTGACCATCTTTCCCAATGC	AGCAAAGCTGCTGGGATATG	135 bp, 62°C
**9**	ELN	GTCCTCCTGCTCCTGCTGT	CTCCTCCTCCAAGGGCTC	127 bp, 62°C
**10**	FN1	AAACCAATTCTTGGAGCAGG	CCATAAAGGGCAACCAAGAG	142 bp, 50°C
**11**	GATA6	TGCAGCAAAAATACTTCCCC	TGTAGAGCCCATCTTGACCC	133 bp, 62°C
**12**	IGF2	GCTTCCAGACACCAATGGGAATCC	TCATATTGGAAGAACTTGCCCACG	364 bp, 60°C
**13**	IGFBP3	AGAGCACAGATACCCAGAACT	TGAGGAACTTCAGGTGATTCAGT	105 bp, 59.5°C
**14**	INHBB	GCGTTTCCGAAATCATCAG	TTTCAGGTAAAGCCACAGGC	134 bp, 59.5°C
**15**	LIMK1	GGAGAGGAAGGAAGCGAGTT	TAGTACTGGTGCGACAGGGA	146 bp, 59.5°C
**16**	PLOD2	GGACTCGGAGAAGCCCTC	CCTTGACCAAGGACCTTCAC	138 bp, 59.5°C
**17**	PTN	TGCAACAAAGGCAGACTGAG	TCCCTGCTTCAGCAGTATCC	148 bp, 59.5°C
**18**	RPL35A	GAACCAAAGGGAGCACACAG	CAATGGCCTTAGCAGGAAGA	236 bp, 58°C
**19**	TAGLN	GCTCTACTGTCTGTTGCCCC	CCTCCAGCTCCTCGTCATAC	135 bp, 62°C
**20**	TGFβ2	AGTGCCTGAACAACGGAT	GTACAAAAGTGCAGCAGG	218 bp, 55°C
**22**	TGM2	TGACCTCCGCAAAGACAAAG	CCAAGTTGCGGAAGCAGTA	241 bp,50°C
**23**	THBS1	CCGGCGTGAAGTGTACTAGCTA	TGCACTTGGCGTTCTTGTT	317 bp, 59°C
**24**	TGFBI	TGTGTGCTGAAGCCATCGTTG	CCGGCTTGTCTGAAAAGGTCA	313 bp,50°C
**25**	TMEPAI	TTCATTCCCTGTCCTCATTGG	GCACAACAGCCATGGAATCA	228 bp, 50°C
**26**	TGFβ1	TCCGAGAAGCGGTACCTGAA	TGCTGTCACAGGAGCAGTGG	266 bp,63.7°C
**27**	TGFβ3	GCGTGAGTGGCTGTTGAGA	CCAAGTTGCGGAAGCAGTA	306 bp,52.7°C
**28**	TGFβRI	TACAGCTTTGCCTGAACTCT	CACGACAGAGTTACCTAAAG	311 bp,52.7°C
**29**	TGFβRII	AGTGTTGGGTTATTGCTAAT	AGTGACTTCACAATGTAAAC	240 bp,54.3°C
**30**	TGFβRIII	ATTCTTTTCAGGCCAGTGGC	TGGAACCTGTATCACAATGGAG	182 bp,63.2°C

### Microarray and data analysis

Whole human genome (4X44 K) oligonucleotide arrays (Agilent Technologies, Santa Clara, USA) were used for microarray experiments. Briefly, 200 ng of RNA from untreated and treated biological duplicate samples (5H; 5 μg/ml areca nut, T; 5 ng/ml TGF-β and 5H+T; 5 μg/ml areca nut with 5 ng/ml TGF-β) were used for the synthesis of cRNA probes labeled with cy3 and cy5 respectively using Low RNA Input Linear Amplification kit (Agilent Technologies, USA) and hybridized according to manufacturer’s protocol. Feature extraction tool version 9.5.3.1 (Agilent Technologies, USA) was used for image analysis. Limma Package from R-bioconductor was used for background subtraction and normalization. Lowess normalization and quantile normalizations were applied to control dye bias and between array technical artifacts. Limma ebayes was applied to access statistically differentially regulated probes [20]. List of differentially regulated probes is based on fold change as calculated by ratios of cy5/cy3 intensity with a cut off of P ≤ 0.05 and fold change ≥1.5.

For hierarchical cluster, Pearson correlation matrix was used as a distance matrix and averaged method for linkage [21]. Gene Set Enrichment Analysis (GSEA) was done as described [22]. Venn diagrams were made using Venny (http://bioinfogp.cnb.csic.es/tools/venny/index.html). The data is submitted to GEO database with accession number GSE59414.

### Collagen contraction assay

The collagen contraction assay was performed with 1.5 x 10^5^ serum deprived hGF cells embedded in collagen gels which were prepared as described [13,23]. Cells were pelleted down and re-suspended in fresh serum free DMEM. Total number of cells was counted and 1.5x10^6^ cells per 600 μl were aliquoted in fresh tubes. Chilled 330 μl of collagen (dissolved in 0.2% acetic acid to a final concentration of 3 mg/ml) was added to the cells and pH was neutralized immediately by the addition of 1M NaOH. This solution was mixed gently and plated into 12 well plates. They were left undisturbed for 2 hours at 37°C to allow gelation of collagen populated with hGF cells. The gels were subsequently detached slowly from the plate using P1000 tip. 1 ml of serum free media was added to each well such that the gels were free floating. Each free floating hGF populated collagen gel was treated with 5 μg/ml of areca nut with or without 5 ng/ml of TGF-β. Decrease in collagen gel diameter was recorded after 24 hours using a ruler along four axis and images were taken under white light in gel documentation system (UviPro platinum, Uvitech UK). Results were plotted as change in total surface area.

### Immunocytochemistry

For immunocytochemistry, 50,000 hGF cells were seeded on cover slips in 12 well plate, serum deprived and treated with 10 μM ALK5 inhibitor (TGFβRI inhibitor, SB 431542, Sigma-Aldrich, USA) two hours prior to 5 μg/ml of areca nut treatment. The treatments with HaCaT condition media are as described earlier. After completion of 48 hours of treatment, hGF cells were fixed and permeabilized using chilled methanol. Residual methanol was washed off with phosphate buffered saline (1X PBS). The protocol for ICC is essentially as described [24]. To block nonspecific staining, cells were incubated with 10% serum for one hour. αSMA antibody was then added at a dilution of 1:150 (ab32575, Abcam, USA) and kept overnight at 4°C. Cells were then washed twice with 1X PBS and incubated with secondary antibody (anti-mouse Alexafluor 488, 1:200 dilution; Invitrogen, USA) for 1 hour at room temperature. Excess secondary antibody was washed off using 1X PBS and the nucleus was stained with propidium iodide (1 mg/ml) for 1–2 minutes. Cover slips were then washed again and mounted in antifade (Invitrogen, Life Technologies, USA). αSMA expression was detected using confocal laser scanning microscope (Ziess, LSM550, apocromat).

### Direct red 80 staining for collagen

Collagen protein was detected by Direct Red 80 dye (Sigma- Aldrich, USA) using previously described protocol [25]. hGF cells were washed with PBS and 1 ml of Bouin’s solution (15:5:1 of aqueous picric acid: 35% formaldehyde: glacial acetic acid) was used as fixative for an hour. Cells were washed with PBS and air dried for 15 minutes followed by addition of 1 ml Direct Red 80 dye (100 mg/ml Direct Red 80 dye prepared in saturated aqueous picric acid solution). Staining was done for 1 hour with mild shaking. After removal of the dye, the excess dye was washed off using 1 ml of 0.01N HCl for 10 minutes. The stained cells were photographed and the dye from cells was extracted using 0.1N NaOH whose optical density (OD) was measured at 550 nm using spectrophotometer (Bio-Rad, SmartSpec-3000 Spectrophotometer). 0.1N NaOH was used as blank and results were plotted as OD/10^5^ cells.

### Statistical analysis

One way analysis of variance (ANOVA) and Benferroni’s Multiple Comparison test was employed to test for significance while making multiple comparisons. Wilcoxon signed rank test was used to compare the significance of median expression values of genes in normal and OSF tissue samples. P value ≤0.05 was considered as significant with P value ≤ 0.01, ≤0.001 and ≤0.0001 represented as *, ** and *** respectively.

## Results

### Areca nut and/or TGF-β induced gene expression profile in human gingival fibroblast (hGF) cells

**Fig 1:**
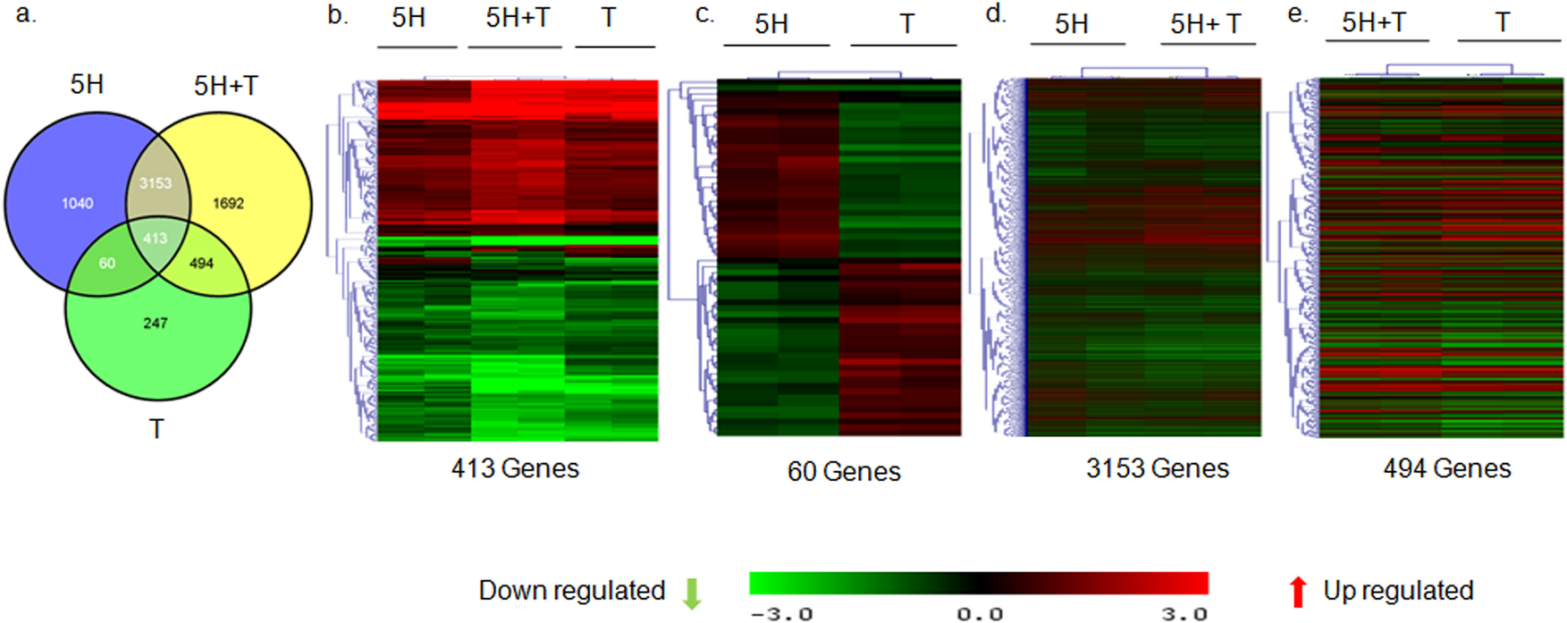
Areca nut and TGF-β induced transcriptome profile in fibroblast (hGF) cells. a] Venn diagram representing differentially regulated genes by areca nut and/or TGF-β in hGF cells. b] Hierarchal cluster of the 413 genes commonly regulated in hGF by areca nut water extract (5H) and areca nut with TGF-β (5H+T). c] Hierarchal cluster of 60 genes oppositely regulated in hGF by areca nut (5H) and TGF-β (T). d] Hierarchal cluster of 3153 genes commonly regulated in hGF by areca nut (5H) and areca nut with TGF-β (5H+T) treatment. e] Hierarchal cluster of genes (494) whose regulation by TGF-β (T) is not influenced by the addition of areca nut (5H). In all hierarchal clusters; red, green and black colours represent up, down and un-regulated genes respectively. The rows represent genes and columns represent various treatments of areca nut on hGF cells (5H; 5 μg/ml) and/or TGF-β (T; 5 ng/ml).

As described in the introduction, areca nut has been proposed as the etiological agent for OSF and has also been shown to influence the activation of TGF-β pathway in epithelial cells [10]. Fibroblasts are the main effectors of fibrosis in OSF. Therefore, decoding the response of fibroblasts to areca nut and TGF-β is essential to understand the molecular mechanisms in the manifestation of OSF. Hence, to delineate the effects of TGF-β along with areca nut on fibroblasts, transcriptome profiling was performed on hGF cells treated with areca nut (5H), TGF-β (T) and areca nut along with TGF-β (5H+T). Data analysis identified 4666 and 1214 differentially regulated genes by areca nut and TGF-β, respectively while areca nut and TGF-β together regulated 5752 genes (Tables 2, 3, 4) as compared to control cells. Venn diagram identified 1040 genes exclusively regulated by 5H as they did not appear in the 5H+T list. Similarly, 247 genes were regulated by T and not by 5H. 5H+T could induce 1692 genes, which were not regulated by either 5H or T. Interestingly, 413 genes were commonly regulated in all the three treatments (Fig 1A).

**Table 2:** List of top 30 up or down regulated genes in hGF cells by areca nut (5H).

Gene Name	Systematic Name	Fold change	GeneName	Systematic Name	Fold Change
TAGLN	NM_001001522	4.6267527	IFIT3	NM_001549	0.12762652
AMAC1L2	NM_054028	4.5002339	XAF1	NM_017523	0.13584186
RNU12	NR_029422	4.4691486	GOLGA4	NM_002078	0.15389305
ATP2B3	NM_021949	4.0558379	CFH	NM_000186	0.15496346
KLF2	NM_016270	4	KTN1	NM_004986	0.15604132
ACTN4	NM_004924	3.8637453	A_23_P3317	A_23_P33173	0.1615441
ENST00000429183	ENST00000429183	3.8370565	IFI44L	NM_006820	0.16266773
PSAT1	NM_058179	3.7842306	SAMD9L	NM_152703	0.16493849
C9orf69	NM_152833	3.732132	HSP90AA1	NM_001017963	0.16724094
DHCR7	NM_001360	3.6300766	NUCB2	NM_005013	0.17924441
CTGF	NM_001901	3.6050019	CENPF	NM_016343	0.18049115
LBH	NM_030915	3.5064229	MME	NM_007289	0.18301071
COTL1	NM_021149	3.5064229	TFPI2	NM_006528	0.18685616
DUSP5P	AK055963	3.4822023	AKAP9	NM_005751	0.19888412
AI042308	AI042308	3.3869812	GBP3	NM_018284	0.20026747
CACNG8	NM_031895	3.3635857	KYNU	NM_003937	0.20447551
LOC100131138	ENST00000331096	3.3172782	TRIP11	NM_004239	0.20447551
PYCR1	NM_006907	3.3172782	PCM1	NM_006197	0.20732989
A_32_P138933	A_32_P138933	3.2943641	ANKRD12	NM_015208	0.20732989
COL1A1	Z74615	3.2716082	IFIT2	NM_001547	0.2102241
FKBP10	NM_021939	3.2716082	LRRN3	NM_018334	0.2102241
HSPB7	NM_014424	3.2042795	GOLGB1	NM_004487	0.21763764
LIMS2	NM_017980	3.1821459	EIF3A	NM_003750	0.22375627
IRF2BP1	NM_015649	3.1821459	IFIH1	NM_022168	0.22375627
ACTG2	NM_001615	3.1601652	TPR	NM_003292	0.22531262
NFIX	NM_002501	3.1383364	NEXN	NM_144573	0.22845786
PHGDH	NM_006623	3.1166583	GBP3	NM_018284	0.22845786
MEX3D	NM_203304	3.0737504	PPIG	NM_004792	0.22845786
DUSP1	NM_004417	3.0525184	LARP7	NM_016648	0.23325825
COL15A1	NM_001855	3.0314331	NOP58	NM_015934	0.23325825

**Table 3:** List of top 30 up or down regulated genes in hGF cells by TGF-β (T).

Gene Name (Up regulated)	Systematic Name	Fold Change	Gene Name (Down regulated)	Systematic Name	Fold Change
IGFBP3	NM_001013398	21.705669	NPTX1	NM_002522	0.01651591
CTGF	NM_001901	19.973289	TFPI2	NM_006528	0.05219299
TPM1	NM_001018004	19.292925	THBD	NM_000361	0.06515411
CRLF1	NM_004750	18.895883	SECTM1	NM_003004	0.07802066
EGR2	NM_000399	18.635737	PTX3	NM_002852	0.09944206
PMEPA1	NM_020182	17.387758	SERPINB2	NM_001143818	0.10013373
FZD8	NM_031866	14.928528	KYNU	NM_003937	0.11265631
COMP	NM_000095	14.123248	COLEC12	NM_130386	0.11265631
AMIGO2	NM_181847	13.361407	NTN1	NM_004822	0.12413656
AK3L1	NM_001005353	12.640661	CCL2	NM_002982	0.13678671
COL4A1	NM_001845	11.080876	WISP2	NM_003881	0.14161049
ID1	NM_002165	10.410735	SLC9A9	NM_173653	0.15604132
HAPLN1	NM_001884	10.410735	BDKRB2	NM_000623	0.15932008
COL8A2	NM_005202	9.4479413	KCNJ2	NM_000891	0.16266773
CSRP2	NM_001321	8.9382971	NR4A3	NM_173198	0.16608573
NOX4	NM_016931	8.6938789	A_32_P23272	A_32_P23272	0.16724094
DACT1	NM_016651	8.3977335	IFIT2	NM_001547	0.17075503
FN1	NM_212482	8.2249106	CXCL2	NM_002089	0.17555561
FNDC1	NM_032532	8.1116758	CCRL1	NM_178445	0.1767767
TAGLN	NM_001001522	8.0556444	ENPP2	NM_006209	0.18174656
SOST	NM_025237	7.94474	SLC14A1	NM_001146037	0.18815584
COL4A2	NM_001846	7.621104	PTGS2	NM_000963	0.18815584
C5orf13	NM_004772	7.5684612	SHC3	NM_016848	0.1907824
LDHA	NM_005566	7.516182	SECTM1	NM_003004	0.19479114
A_23_P123234	A_23_P123234	7.3615012	C3	NM_000064	0.19888412
PSAT1	NM_058179	6.5887281	SEMA3A	NM_006080	0.20877198
PGK1	NM_000291	6.5887281	PHLDA1	NM_007350	0.20877198
BNIP3	NM_004052	6.408559	EDNRB	NM_003991	0.21168633
LMCD1	NM_014583	6.0628663	KRT32	NM_002278	0.21315872
C10orf10	NM_007021	6.0628663	NOV	NM_002514	0.21464136

**Table 4:** List of top 30 up or down regulated genes in hGF cells by areca nut and TGF-β (5H+T).

Gene Name	Systematic Name	Fold Change	Gene Name	Systematic Name	Fold Change
EGR2	NM_000399	29.242606	NPTX1	NM_002522	0.03280365
FZD8	NM_031866	22.943284	TFPI2	NM_006528	0.05183247
COMP	NM_000095	21.856644	COLEC12	NM_130386	0.08362047
CRLF1	NM_004750	20.39297	MMP1	NM_002421	0.0842021
IGFBP3	NM_001013398	20.112214	XAF1	NM_017523	0.08838835
PMEPA1	NM_020182	18.126142	IFIT3	NM_001549	0.09407792
ID1	NM_002165	14.825409	IFIT2	NM_001547	0.10366494
CTGF	NM_001901	14.723002	CCL2	NM_002982	0.10511205
A_24_P887857	A_24_P887857	14.520306	KYNU	NM_003937	0.10806715
KRT17	NM_000422	13.642158	CFH	NM_000186	0.10957572
COL4A1	NM_001845	12.640661	HSP90AA1	NM_001017963	0.11990801
AK3L1	NM_001005353	12.553346	MME	NM_007289	0.12074204
LRRC15	NM_130830	11.63178	PTX3	NM_002852	0.12074204
DACT1	NM_016651	11.392402	PTN	NM_002825	0.12242754
KRT14	NM_000526	10.777869	CASP1	NM_033292	0.12674493
COL8A2	NM_005202	10.267407	SERPINB2	NM_001143818	0.12674493
FSTL3	NM_005860	9.6464626	KTN1	NM_004986	0.12851423
ENST00000300992	ENST00000300992	9.3826796	CENPF	NM_016343	0.13121459
LMCD1	NM_014583	9.1261097	REV3L	NM_002912	0.13212726
AMIGO2	NM_181847	8.8765558	DCN	NM_001920	0.13773814
C10orf10	NM_007021	8.7543496	CXCL1	NM_001511	0.14063231
IL11	NM_000641	8.6338259	KGFLP1	NR_003674	0.14259546
TAGLN	NM_001001522	8.3397261	GOLGA4	NM_002078	0.14259546
CR597597	CR597597	8.2249106	BIRC3	NM_001165	0.14358729
FNDC1	NM_032532	7.7812396	GBP3	NM_018284	0.14762408
MT3	NM_005954	7.621104	BEX2	NM_032621	0.14865089
PSAT1	NM_058179	7.4642639	FGF7	NM_002009	0.14865089
AI042308	AI042308	7.4127045	THBD	NM_000361	0.14968484
A_23_P123234	A_23_P123234	7.3615012	SAMD9L	NM_152703	0.14968484
SOST	NM_025237	7.0128458	IFIH1	NM_022168	0.15283003

Hierarchal clustering of these 413 genes revealed that 5H+T profile clustered in between 5H and T thereby signifying similarity in the expression of these genes by 5H+T treatment with either 5H or T profiles. Moreover, the expression of most of these genes seemed to be enhanced by the combined treatment with areca nut and TGF-β (5H+T) (Fig 1B). Intriguingly, there were 60 genes which showed opposite regulation by 5H or T treatments and hence they did not appear in the profile of 5H+T treatment (Fig 1A and 1C). These genes may not have any implication in OSF. The 3153 genes regulated by 5H but not by T (Fig 1A) could be exclusive targets of 5H as they were not potentiated by 5H+T (Fig 1D). Similarly, 494 genes which were TGF-β targets but not regulated by areca nut (Fig 1A) were not potentiated in 5H+T treatment implying that these were essentially TGF-β targets (Fig 1E).

### Areca nut induces different gene expression profiles in fibroblast and epithelial cells

**Fig 2:**
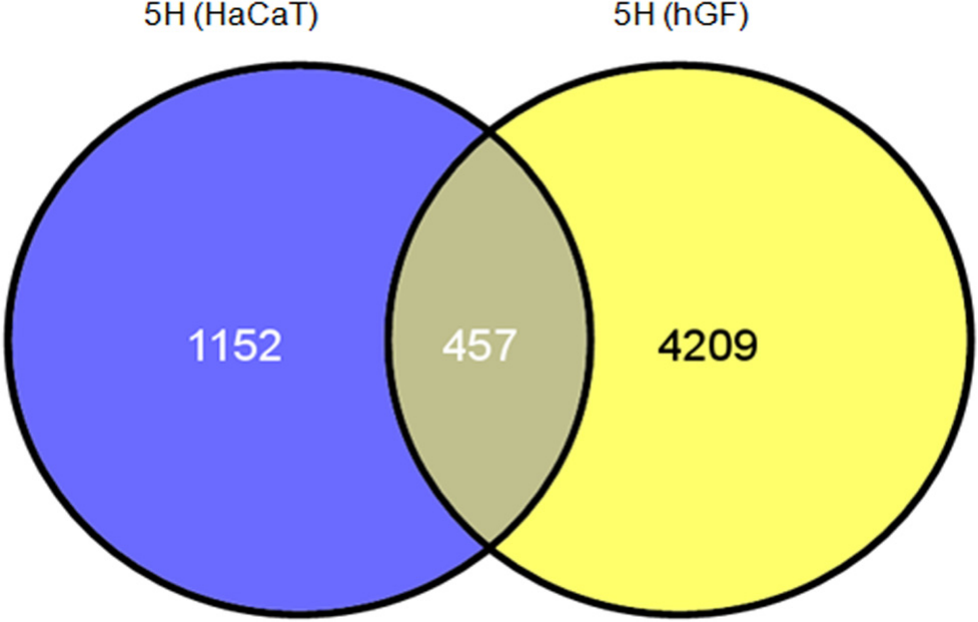
Areca nut induces different gene expression profiles in fibroblast and epithelial cells. Venn diagram representation of genes regulated by areca nut in epithelial (HaCaT, GSE 38227) and fibroblast cells (hGF). 457 genes are commonly regulated by areca nut in both the cell types while bulk of the differentially regulated genes (1152 in HaCaT and 4209 in hGF) are mutually exclusive.

We previously reported the transcriptome profile induced by areca nut in epithelial cells [10]. To further explore whether areca nut actions are similar on epithelial (HaCaT) and fibroblast (hGF) cells; expression profiles of genes regulated by areca nut in these cell types were compared. Analysis revealed 457 commonly regulated genes by areca nut in both HaCaT and hGF cells while regulation of 1152 genes in HaCaT and 4209 genes in hGF was non-overlapping (Fig 2). This indicates that areca nut induces differential transcriptome profiles in epithelial and fibroblast cells.

### Transcriptome profile of OSF shares similarity with areca nut and TGF-β regulated profiles in HaCaT and hGF

**Fig 3:**
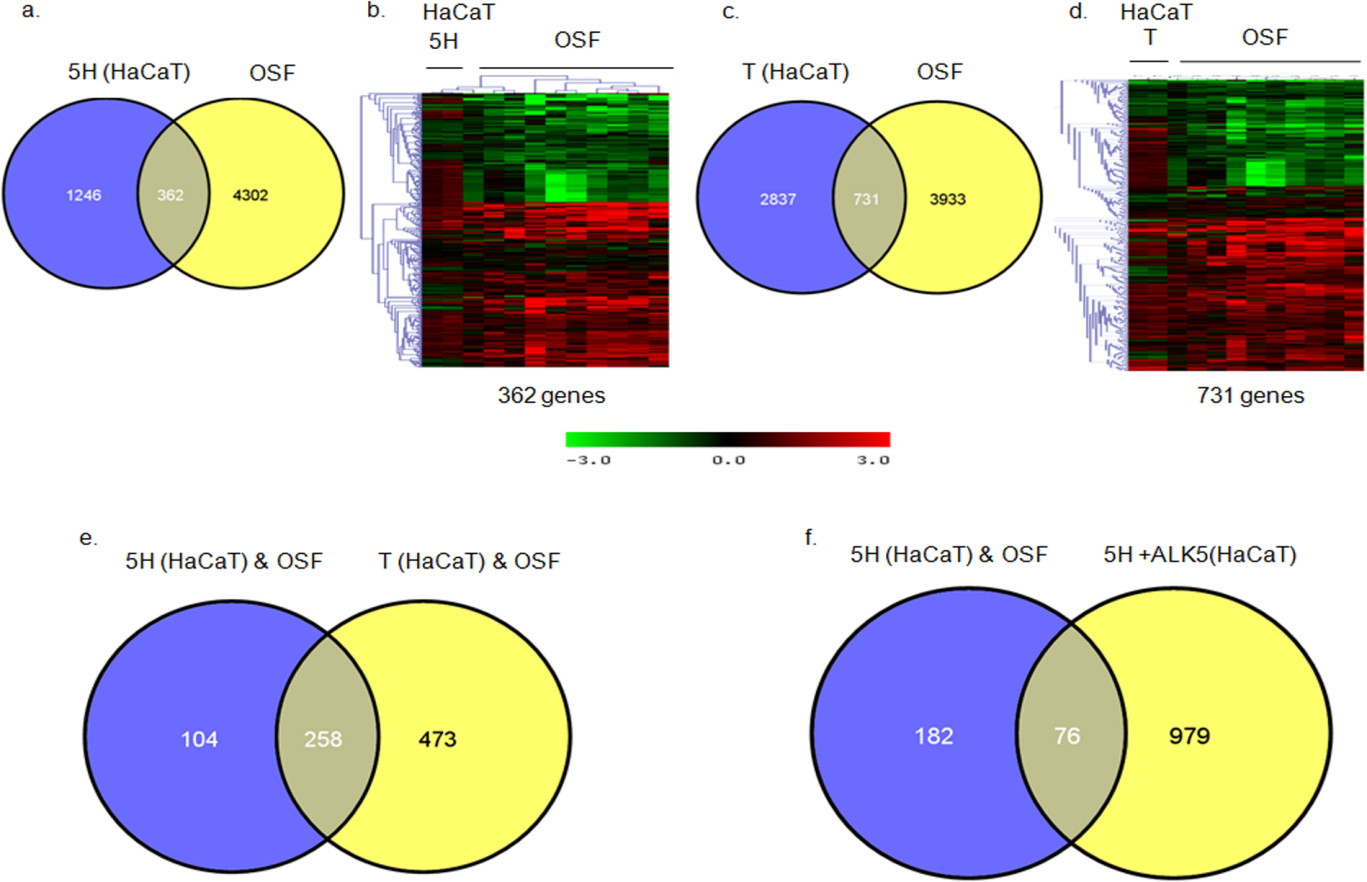
Genes regulated by areca nut in epithelial cells and OSF are via TGF-β. Previously published transcriptome profile of OSF tissues (GSE 20170) [6] was compared with that of areca nut and/ or TGF-β regulated transcriptome in HaCaT (GSE 38227) [10] and hGF cells. a & b] Venn diagram and hierarchal cluster representing distribution of differentially or commonly regulated genes by areca nut in HaCaT cells and OSF. c & d] Venn diagram and hierarchal cluster representing distribution of differentially or commonly regulated genes by TGF-β in HaCaT cells and OSF. e] Venn diagram represents the 252 genes common between the 362 (252+104) genes regulated by areca nut in HaCaT and OSF and the 731 (252+ 473) genes regulated by TGF-β in HaCaT cells and OSF. f] Out of the 252 genes discussed in 3e, 182 genes are not regulated by areca nut in presence of ALK5 inhibitor (SB 431542) in HaCaT cells. This indicates that these 182 genes are regulated by areca nut are via TGF-β in HaCaT cells and possibly in OSF. In all hierarchal clusters; red, green and black colours represent up, down and un-regulated genes respectively. The rows represent genes and columns represent OSF and various treatments of areca nut (5H; 5 μg/ml) and/or TGF-β (T; 5 ng/ml).

Since our data indicated differential response of both cell types to areca nut, we hypothesized that the transcriptome profile of OSF tissues is a combination of areca nut response of both epithelial and fibroblast cells. Therefore, the previously published microarray data of OSF tissues [6] was compared with that of areca nut and/ or TGF-β regulated gene expression in HaCaT [10] and hGF cells. The data analysis suggests that majority of the genes regulated by areca nut in HaCaT cells and common with OSF are TGF-β targets (Fig 3A–3F).

**Fig 4:**
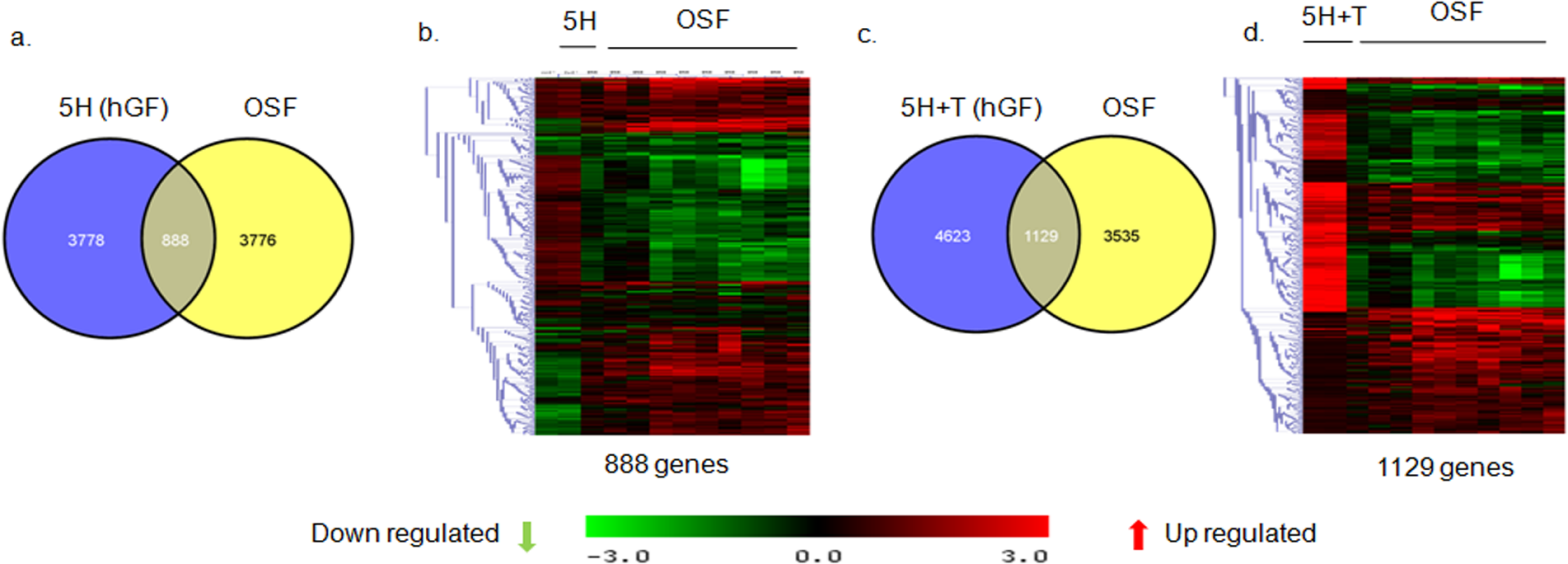
Areca nut and TGF-β induced transcriptome profile in fibroblast (hGF) cells is similar to OSF. a & b] Venn diagram and hierarchal cluster representing genes commonly or differentially regulated by areca nut in hGF cells and OSF. c & d] Venn diagram and hierarchal cluster representing genes commonly or differentially regulated in hGF by areca nut with TGF-β (5H+T) and OSF. In all hierarchal clusters; red, green and black colours represent up, down and un-regulated genes respectively. The rows represent genes and columns represent OSF and various treatments of areca nut (5H; 5 μg/ml) and/or TGF-β (T; 5 ng/ml).

Among the 4666 genes differentially regulated by areca nut in hGF cells; 888 were common with genes regulated in OSF compared to normal tissues (Fig 4A and 4B). Upon areca nut and TGF-β treatment of hGF cells and comparison with differentially regulated genes in OSF, the number of common genes increased to 1129 (Fig 4C and 4D). This suggests combined actions of areca nut and TGF-β on fibroblasts are important in the disease process.

### Validation of areca nut and TGF-β regulated genes in hGF

**Fig 5:**
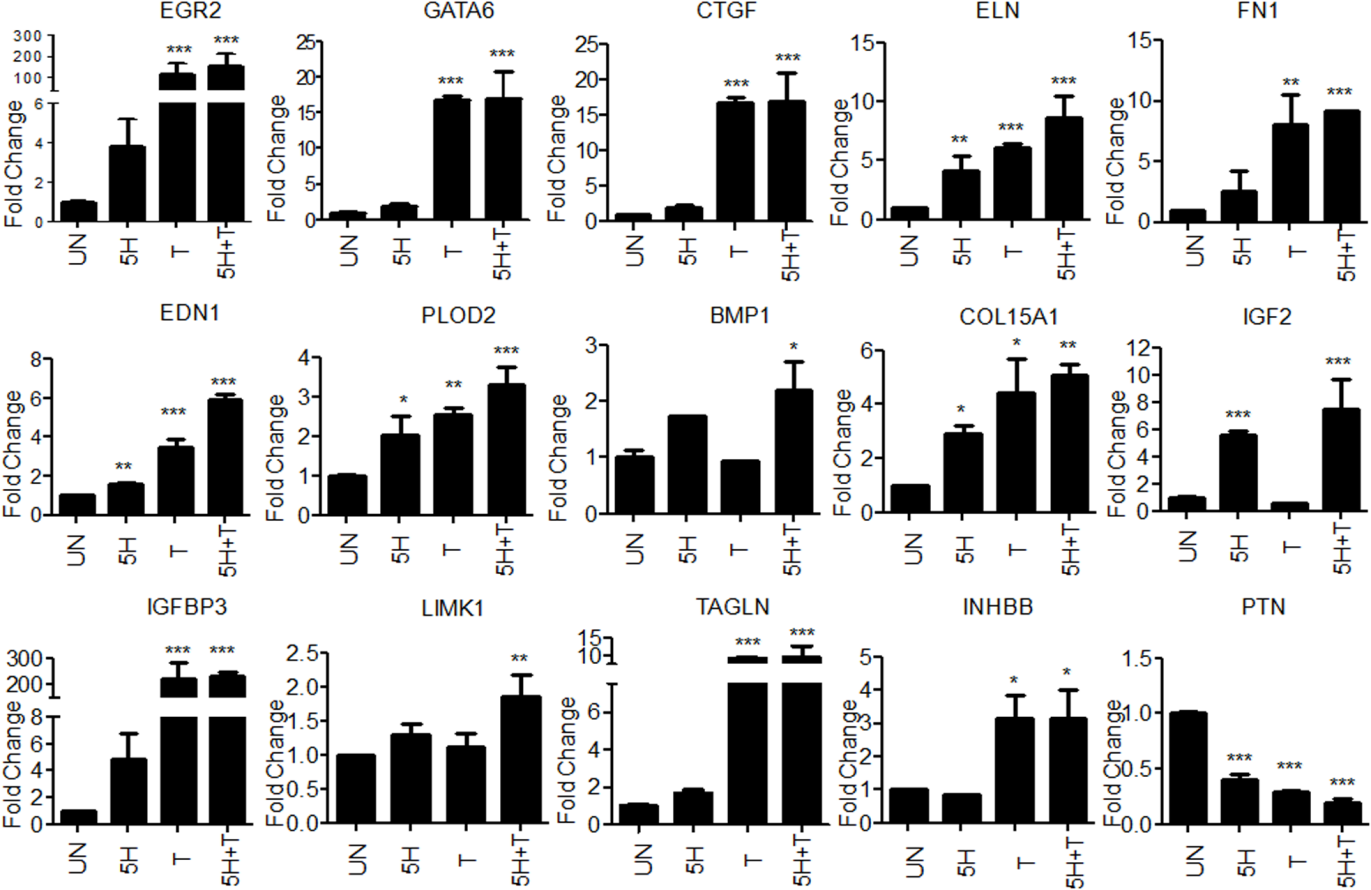
Validation of areca nut and TGF-β regulated genes in hGF cells. Serum starved hGF cells were treated with areca nut water extract (5H; 5 μg/ml), TGF-β (T; 5 ng/ml) and both together (5H+T) for 72 hours (n = 3) followed by the study of gene expression changes using qPCR. Y-axis represents the fold changes of each treatment. P values ≤ 0.0001, ≤ 0.001, ≤ 0.01 are depicted as ***, ** and * respectively.

Some of the differentially expressed genes in OSF [10] which were also found to be regulated in hGF cells by areca nut and/or TGF-β were selected for validation by qPCR (Fig 5). Connective tissue growth factor (CTGF), Endothelin (EDN1) and Fibronectin 1(FN1) are over expressed and implicated in OSF pathogenesis [4,6,26]. These genes are significantly up regulated by areca nut and TGF-β in hGF cells. Similarly, other genes like Early Growth response protein 2 (EGR2), GATA binding protein 6 (GATA6), Collagen 15A1 (COL15A1), Bone morphogenetic protein 1 (BMP1), Procollagen-lysine,2-oxoglutarate 5-dioxygenase 2 (PLOD2), LIM domain kinase 1 (LIMK1), Transgelin (TAGLN), Inhibin beta B (INHBB); Insulin growth factor 2 (IGF2), Insulin growth factor binding protein 3 (IGFBP3), Endosialin (CD248) and Pleiotrophin (PTN) were also validated as areca nut and TGF-β targets.

### Gene Set Enrichment Analysis (GSEA) of differentially regulated genes by areca nut and TGF-β revealed pathways that are common with OSF

GSEA was performed to further explore whether areca nut and TGF-β regulated pathways in hGF were common with the previously reported differentially regulated pathways in OSF [6]. Interestingly, all the positively enriched pathways by areca nut and TGF-β in hGF cells were differentially regulated in OSF also (Table 5). These pathways may have important role in OSF manifestation. In contrast none of the negatively enriched pathways in hGF were differentially regulated in OSF (Table 5) and therefore, may not contribute to OSF pathology. Hence, this corroborated the role of areca nut and TGF-β in manifesting fibrotic phenotype.

**Table 5:** Gene Set Enrichment analysis of differentially regulated genes in hGF cells by areca nut and TGF-β.

S. No.	Positively Enriched Pathways	Number of Genes	S. No.	Negatively Enriched Pathways	Number of Genes
1.	Focal Adhesion	**114**	1.	Spliceosome	**86**
2.	Neuroactive Ligand Receptor Interaction Pathway	**99**	2.	Ribosome	**56**
3.	Hypertrophic Cardiomyopathy HCM	**51**	3.	NOD Like Receptor Signaling Pathway	**40**
4.	Dilated Cardiac Myopathy	**49**	4.	Antigen Processing and Presentation	**33**
5.	Gap Junction	**47**	5.	RIG I Like Receptor Signaling Pathway	**28**
6.	Arrythmogenic Right Ventricular Cardiomyopathy ARVC	**44**	6.	Proteasome	**28**
7	ECM Receptor Interaction	**42**	7	Complement and Coagulation Cascades	**26**
8	Glycolysis Gluconeogenesis	**33**	8	Cytosolic DNA Sensing Pathway	**20**
9	Basal Cell Carcinoma	**24**			
10	Fructose and Mannose Metabolism	**19**			
11	Pentose Phosphate Pathway	**16**			

### Areca nut enhances TGF-β mediated fibroblast activation

**Fig 6:**
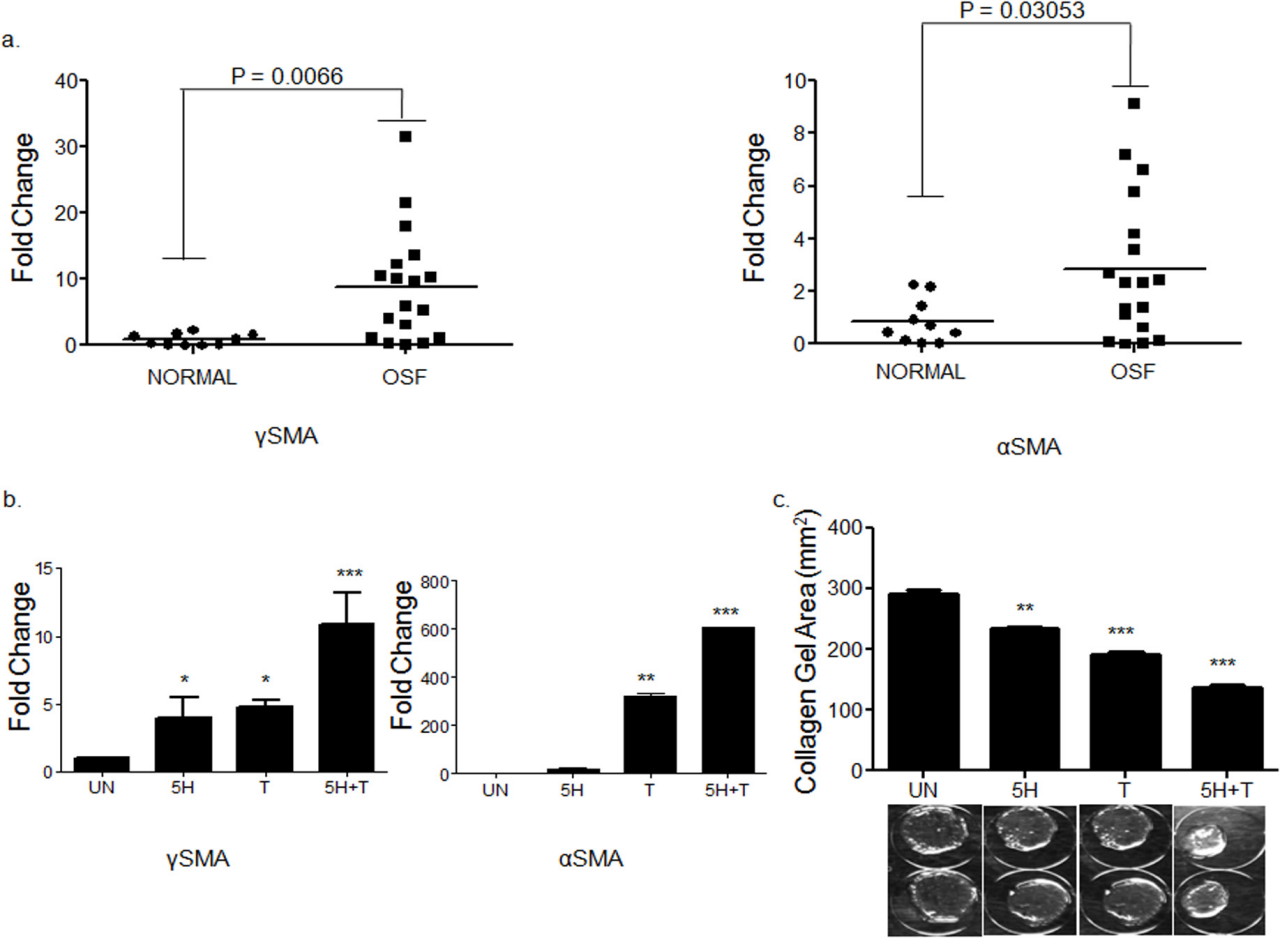
Areca nut enhances TGF-β mediated fibroblast activation. a] Scatter plots of qPCR evaluation of myofibroblast markers αSMA and γSMA in normal and OSF tissues. Each square represents expression in one sample and horizontal line represents the median expression. Both the genes are up regulated in OSF as compared to normal tissues. P values calculated using Wilcoxon signed rank test are < 0.0066 for γSMA and 0.03053 for αSMA. b] qPCR analysis of γSMA and αSMA regulation by areca nut and/or TGF-β in hGF cells. c] Estimation of collagen contraction of hGF populated collagen gel upon areca nut and/or TGF-β treatment. Areca nut (5H), TGF-β (T) and areca nut together with TGF-β (5H+T) decrease the collagen surface area by 233.599 mm^2^, 191.663 mm^2^ and 136.633 mm^2^ respectively as compared to control (290.941 mm^2^). Areca nut and TGF-β together (5H+T) decrease the surface area of collagen gel significantly more than areca nut (5H) or TGF-β (T) alone. Representative images of the decrease in surface area by each of the treatments are given below the graph. P values ≤ 0.0001, ≤ 0.001, ≤ 0.01 are depicted as ***, ** and * respectively. UN- untreated, areca nut- 5H; 5 μg/ml, T–TGF-β; 5 ng/ml.

Fibroblast activation is a hall mark of fibrotic disorders. We therefore confirmed the over expression of myofibroblast markers Alpha smooth muscle actin (αSMA) and Gamma smooth muscle actin (γSMA) in OSF patients (Fig 6A). In light of the observation of areca nut and TGF-β induced profile in hGF being similar to OSF, we studied the activation of hGF cells by a combination of both areca nut and TGF-β. Both αSMA and γSMA expression was more by areca nut and TGF-β treatment than with each of them alone (Fig 6B). Collagen contraction assay was performed to confirm that this potentiation in the expression of myofibroblast markers by areca nut and TGF-β translated into a stronger contractile phenotype. Areca nut along with TGF-β decreased the surface area of hGF populated collagen gels to a significantly greater extent than areca nut or TGF-β alone (Fig 6C). Therefore, TGF-β and areca nut together could enhance fibroblast activation thereby increasing contractility of the cells which is also implicated in OSF [13].

### Areca nut actions on fibroblasts are enhanced by epithelial mesenchymal interaction via TGF-β

Areca nut has been shown to induce TGF-β in epithelial cells [10] and also enhances fibroblast activation in combination with TGF-β invoking a possible epithelial mesenchymal interaction in the initiation of OSF. Therefore, to test this possibility *in-vitro*, HaCaT cells were treated with areca nut with or without ALK5 inhibitor (TGFβRI inhibitor). The condition medium of these cells was used to treat serum deprived hGF cells to study the effect of epithelial factors induced by areca nut, in particular TGF-β.

**Fig 7:**
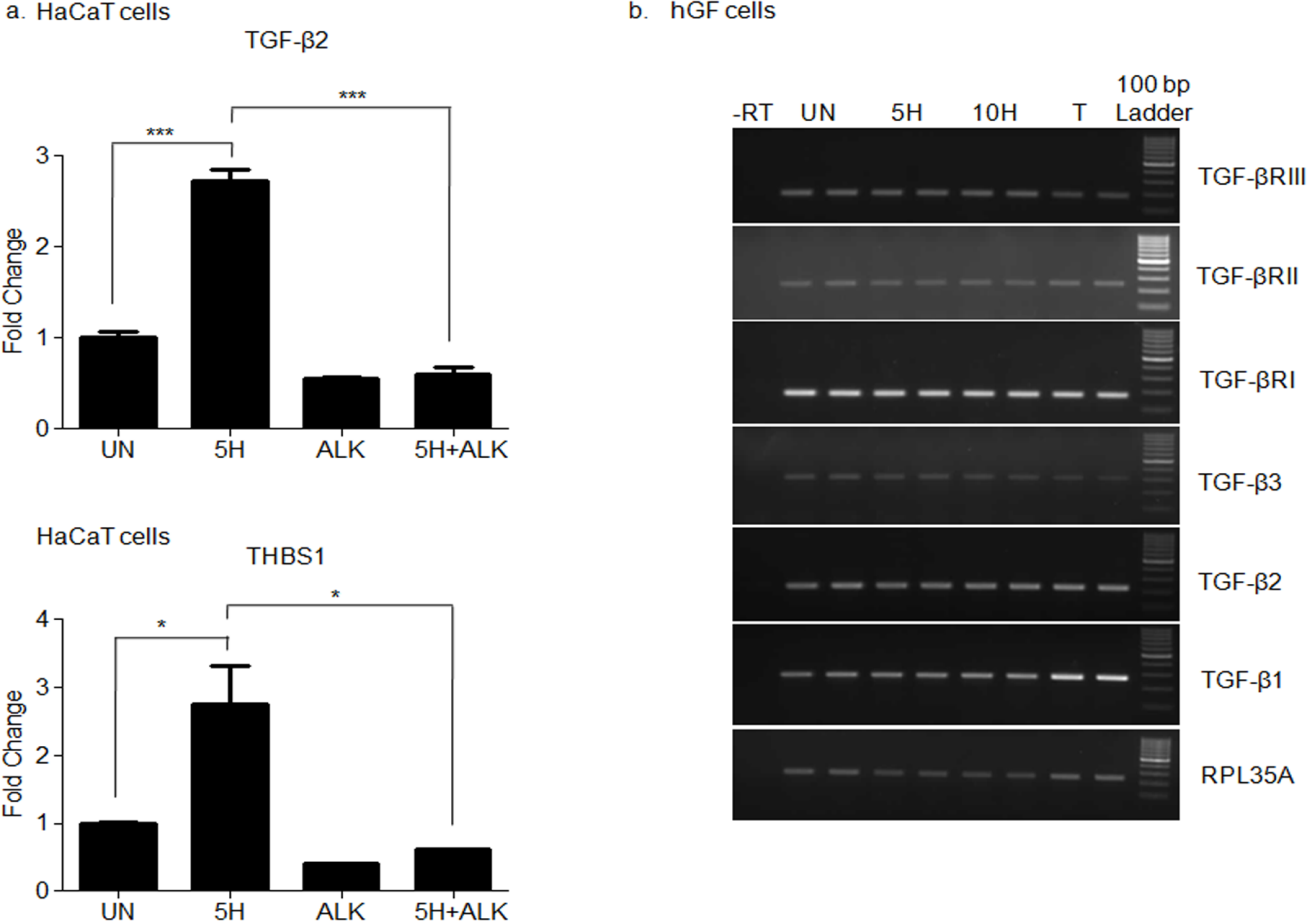
Areca nut induces TGF-β in HaCaT but not in hGF cells. a] Serum deprived HaCaT cells were treated with areca nut (5H; 5 μg/ml) with or without ALK5 inhibitor (10 μM of SB 431542) as described in material and methods. Expression of TGF-β and its activator THBS1 was analyzed by qPCR. Areca nut induced TGF-β and THBS1 which was compromised by ALK5inhibitor (SB 431542) P values ≤0.0001, ≤0.001, ≤0.01 are depicted as *** ** and * respectively. b] Serum deprived hGF cells were treated with areca nut and TGF-β (5H; 5 μg/ml; 10H; 10 μg/ml; TGF-β; T; 5 ng/ml). Expression of TGF-β ligands and receptor isoforms were assessed by semi quantitative PCR. Areca nut treatment did not induce TGF-β ligands and receptor isoforms. TGF-β treatment induced TGF-β1 isoform in hGF cells.

Qunatitative RT-PCR was performed to confirm the induction of TGF-β by areca nut and compromised by ALK5 inhibitor in HaCaT cells (Fig 7A). PCR studies were performed on areca nut treated hGF cells to evaluate the expression of TGF-β ligands and receptors. Treatment of areca nut on hGF cells did not induce any of the three TGF-β ligands nor influenced the expression of TGF-β receptor isoforms (Fig 7B). This also corroborates with the microarray data of hGF cells treated with areca nut wherein none of the TGF-β ligands or receptors were found to be regulated by areca nut in hGF cells.

**Fig 8:**
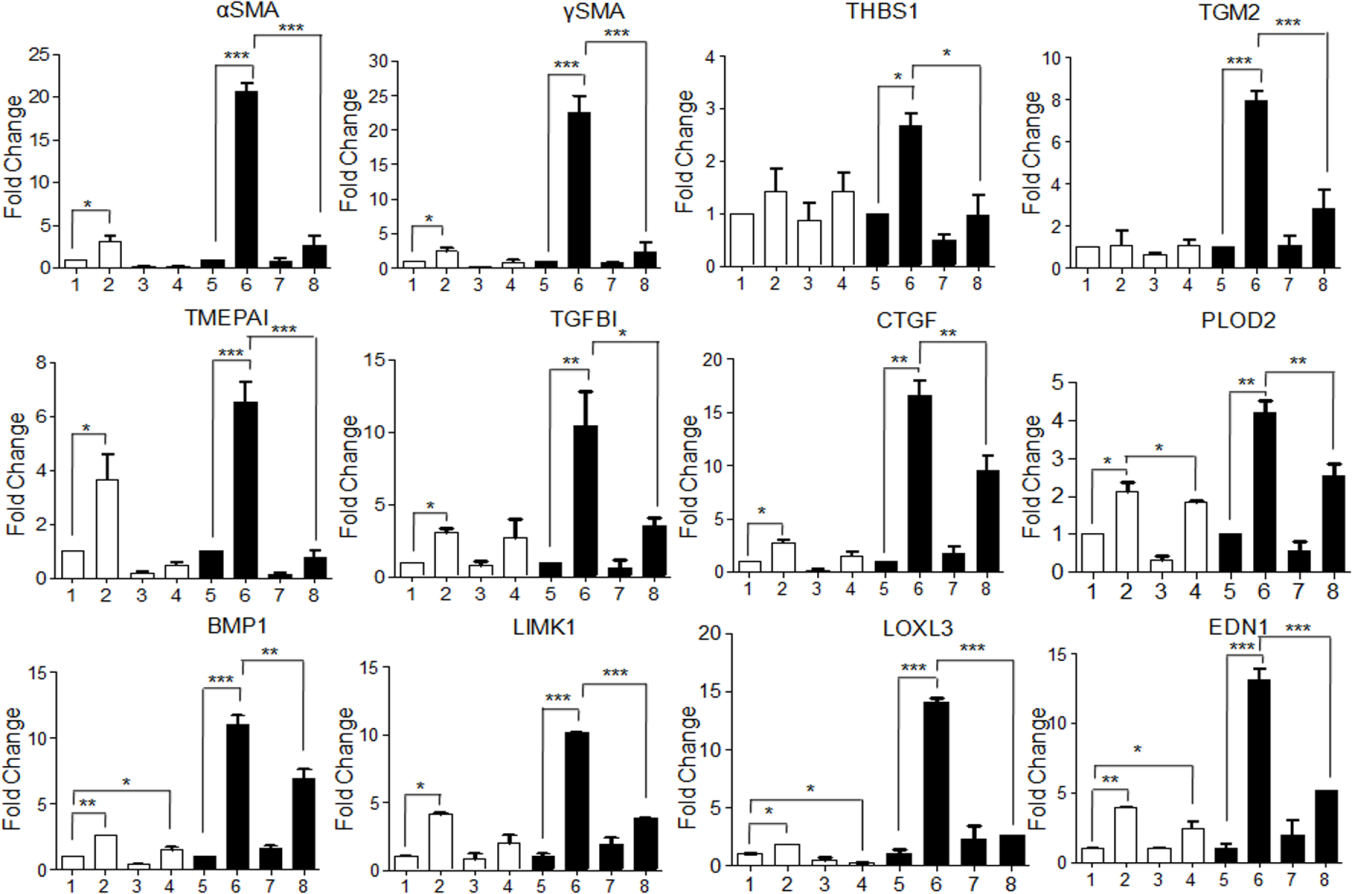
Areca nut actions on fibroblasts are enhanced by epithelial mesenchymal interaction via TGF-β. To study the epithelial mesenchymal interaction, confluent cultures of HaCaT cells were serum starved for 24 hours followed by 10 μM ALK5 inhibitor (TGFβRI inhibitor, SB 431542, Sigma-Aldrich, USA) treatment 2 hours prior to areca nut treatment (5 μg/ml). Meanwhile hGF cells were serum deprived for 24 hours such that the treatment time point coincided with completion of 48 hour treatment on HaCaT cells. At this time point, the condition medium of areca nut (with or without ALK5 inhibitor; SB 431542) treated HaCaT cells was transferred to hGF cells and simultaneously direct treatment of areca nut with or without ALK5 inhibitor; SB 431542 was also performed and both were maintained for 48 hours and gene expression was studied by qPCR. The bar diagrams represent regulation of αSMA/ACTA2, γSMA/ACTG2, THBS1, TGM2, TMEPAI, TGFBI, CTGF, PLOD2, BMP1, LIMK1, LOXL3 and EDN1 in hGF cells upon treatment with 1- untreated; 2- areca nut, 3- ALK5 inhibitor(SB 431542), 4- areca nut with ALK5 inhibitor (SB 431542) (white bars), 5- condition media of untreated HaCaT cells, 6- condition media of areca nut treated HaCaT cells, 7- condition media of ALK5 inhibitor (SB 431542) treated HaCaT cells and 8- condition media of areca nut with ALK5 inhibitor (SB 431542)treated HaCaT cells (black bars). P values <0.0001, <0.001, <0.01 are depicted as ***, ** and * respectively.

**Fig 9:**
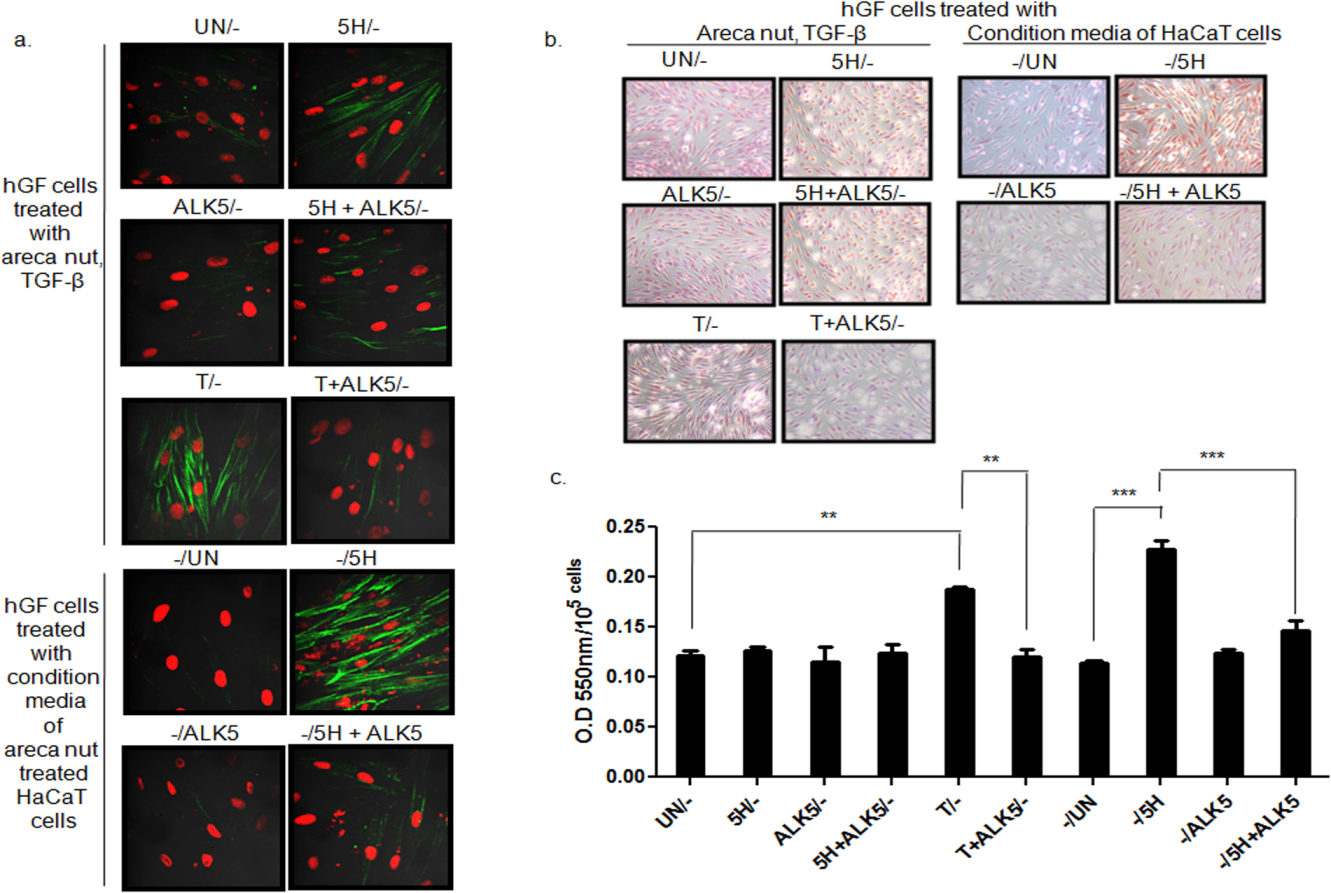
Epithelial secretome induces fibroblast activation and collagen via TGF-β. Condition medium of HaCaT cells treated with areca nut with or without ALK5 inhibitor; SB 431542 (-/UN; condition media of untreated HaCaT cells,-/5H, areca nut treated HaCaT cells,-/ALK5; ALK5 inhibitor treated HaCaT cells and-/5H+ALK5 inhibitor treated HaCaT cells was used to treat serum deprived hGF cells for 48 hours. Simultaneous direct treatment of areca nut with or without ALK5 inhibitor (SB 431542) (UN/-, 5H/-, ALK5/-, 5H+ALK5/-) was given to another set of serum deprived hGF cells for the same duration. Fibroblast activation and total collagen was assessed by αSMA stress fiber formation by immunocytochemistry and direct red 80 staining respectively. a] Condition media of areca nut treated HaCaT cells (-/5H) induced αSMA stress fibres significantly more as compared to untreated (-/UN) and direct treatment of hGF cells with areca nut (5H/-). It got compromised with ALK5 inhibitor, SB 431542 (-/ALK5). Direct treatment of TGF-β with (T+ALK/-) or without ALK5 inhibitor (T/-) was used as positive control for the experiment (image magnification 63X). b] Representative images for total collagen staining by direct red 80 (image magnification 10X) expressed in hGF cells upon respective treatments depicted above each panel. The treatments are as described in 4a. Note the significant increase in the collagen staining when hGF cells were treated directly with TGF-β or conditioned media of HaCaT cells treated with areca nut. Both these regulations were compromised in the presence of ALK5 inhibitor (SB 431542). c] Bar diagram showing quantitation of direct red staining for total collagen measured as O.D per 10^5^ cells of treatments described in 4b.

Treatment of hGF with conditioned medium of areca nut treated HaCaT cells induced αSMA, γSMA, Thrombospondin 1 (THBS1); Transglutaminase 2 (TGM2); Transmembrane prostrate androgen-induced protein (TMEPAI); Transforming growth factor β induced (TGFBI); CTGF; PLOD2; BMP1; LIMK1; LOXL3 and EDN1 to a significantly higher extent than control. This induction was compromised by ALK5 inhibitor highlighting the involvement of TGF-β in the secretome (Fig 8). Interestingly, over expression of these genes has been observed in OSF [6]. To corroborate this, staining for αSMA and total collagen was performed on hGF cells following the same treatment protocol. Areca nut induced factors in HaCaT cells increased the protein expression of αSMA and total collagen more than that of control and direct treatment of areca nut or TGF-β on hGF cells. This increase was obliterated in the presence of ALK5 inhibitor (Fig 9A, 9B and 9C). This is in line with the mRNA expression data discussed earlier.

Taken together, these data suggest that areca nut induced secretory factors by HaCaT cells are able to induce myofibroblast phenotype akin to OSF. Moreover, TGF-β is responsible for this phenotype in the areca nut induced secretome.

### Epithelial factors maintain basal expression of pro-fibrotic genes in fibroblasts

**Fig 10:**
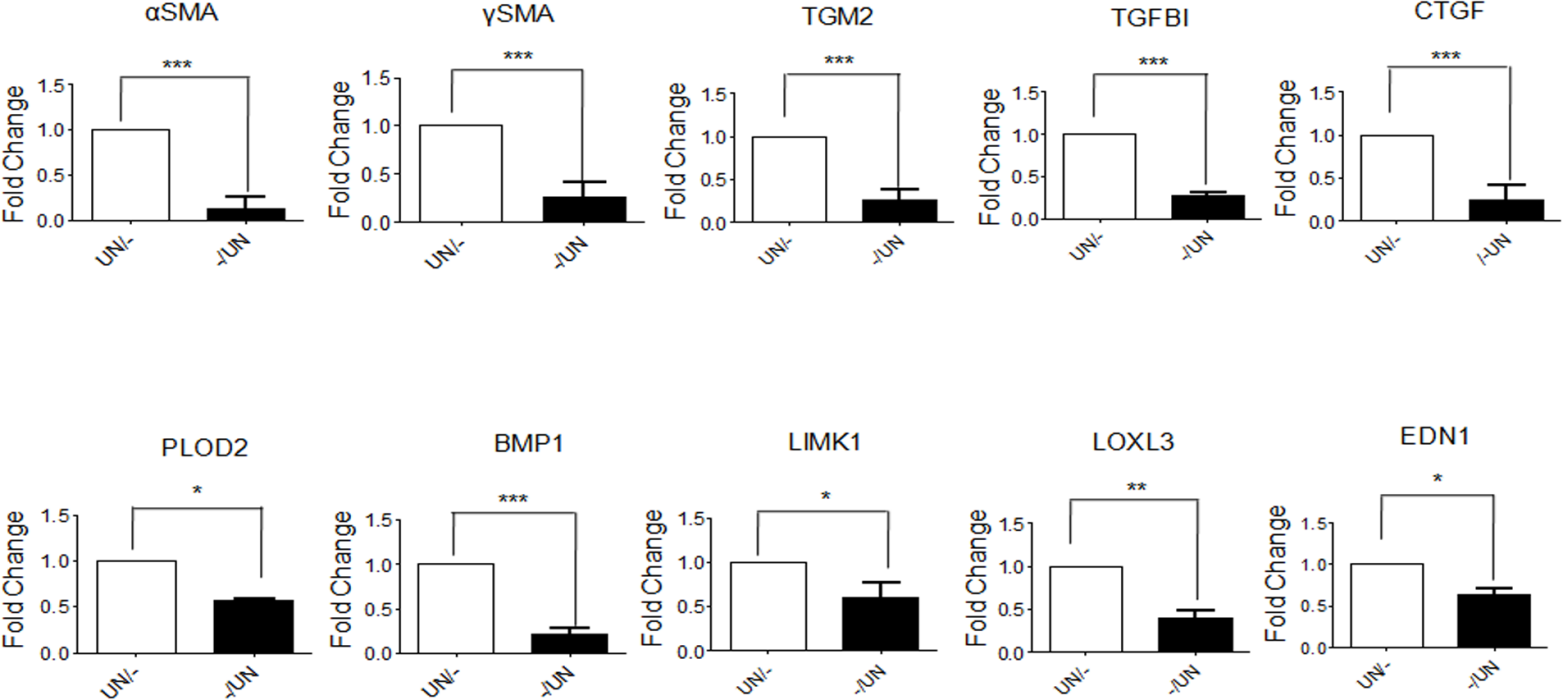
Epithelial factors maintain basal expression of pro-fibrotic genes in fibroblasts. Condition medium of untreated HaCaT cells (-/UN, white bars) was used to treat serum deprived hGF cells for 48 hours. Simultaneously, hGF cells were maintained in serum free medium as control (UN/-, black bars). Expression of αSMA/ACTA2, γSMA/ACTG2, TGM2, TGFBI, CTGF, PLOD2, BMP1, LIMK1, LOXL3 and EDN1 genes significantly decreased upon treatment with condition medium of untreated HaCaT cells.

Comparison of the basal expression of fibroblast activation markers in hGF cells cultured in serum deprived medium and in conditioned medium of untreated HaCaT cells was also done. Expression of pro fibrotic genes αSMA, γSMA, TGM2, TGFBI, CTGF, PLOD2, BMP1, LIMK1, LOXL3 and EDN1 were found to be down regulated in hGF cells treated with condition medium of untreated HaCaT cells (Fig 10). These observations suggest a role for epithelium in maintaining the normal fibroblast phenotype.

## Discussion

Oral submucous fibrosis (OSF) is a condition affecting habitual chewers of areca nut. Our previous work has indicated that areca nut extract as well as its alkaloid and polyphenol fractions induce TGF-β in epithelial cells [10]. Hence, we hypothesized that fibroblasts may respond not only to areca nut but also to TGF-β, to attain a phenotype similar to OSF. In tune with this, transcriptome profiles suggested that areca nut and TGF-β together potentiate the regulation of genes in human gingival fibroblast (hGF) cells.

Epithelial atrophy and increase in fibroblast population along with deposition of excess extra cellular matrix are hallmarks of OSF [2]. In line with this, differential response of epithelial and fibroblast cells to areca nut also implied that these cell types play different roles in the disease process. Additionally, both epithelium and fibroblast cells can be implicated in OSF manifestation as areca nut and TGF-β regulated transcriptome profiles of HaCaT and hGF cells overlapped significantly with OSF profile. Moreover, areca nut and TGF-β were found to enrich pathways in hGF cells which are differentially regulated in OSF; notably metabolic and matrix associated pathways. They also regulated the expression of pro-fibrotic growth factors; CTGF, FN1, EDN1, collagen stabilizing and maturation genes; PLOD2, BMP1 and cytoskeletal reorganizing genes; LIMK1 and TAGLN and transcription factors GATA6, EGR2 in fibroblasts. EGR2 is reported to mediate pro-fibrotic actions of TGF-β in pulmonary fibrosis [27]. Expression of all these genes may have important implications in the progression of OSF.

Our study revealed that areca nut and TGF-β can confer enhanced contractile phenotype (hallmark of fibroblasts in various fibrotic disorders) as well as induce myofibroblast markers αSMA and γSMA in hGF cells. The expression of γSMA in OSF has not been reported and TGF-β is known to induce γSMA in prostrate myofibroblasts [28].

Our data also highlights that direct treatment of areca nut does not regulate TGF-β ligands and receptors in hGF cells. This is in line with our previously published report that areca nut does not induce pSMAD2 (read out of activated TGF-β signaling) in hGF cells [10]. In addition, we provide proof of epithelial- mesenchymal interaction which is mediated via TGF-β induced by areca nut in epithelial cells. This suggests that areca nut induced secretory factors by HaCaT cells could activate fibroblasts and induce genes which play important roles in manifestation of OSF. Corroborating these data; areca nut induced secretome by HaCaT cells also increased protein expression of αSMA and collagen which was abrogated with ALK5 inhibitor providing further evidence of TGF-β’s contribution. This is similar to the role of injured epithelium and epithelial cell- fibroblast interaction in the manifestation of pulmonary and liver fibrosis [29,30]. Arecoline has also been shown to injure epithelial cells via ROS induction and induce cell cycle arrest [31]. In light of our data and these studies, we propose that constant injury inflicted by areca nut and its constituents to the epithelium may drive OSF.

Down-regulation of genes in the fibrosis pathway when hGF cells treated with condition medium of untreated HaCaT cells is intriguing. This suggests that epithelial cells can suppress an inherent capability of fibroblasts to activate the fibrotic/wound repair program. This is corroborated by the report that mouse lung epithelial cell derived secretory factors can suppress fibroblast growth whereas bleomycin mouse model derived epithelial cell factors promote growth of fibroblasts *in vitro* [32]. Further studies are needed to identify the factor (s) responsible for the suppression of fibrosis related genes, which may lead to a potential therapeutic target.

## Conclusion

**Fig 11:**
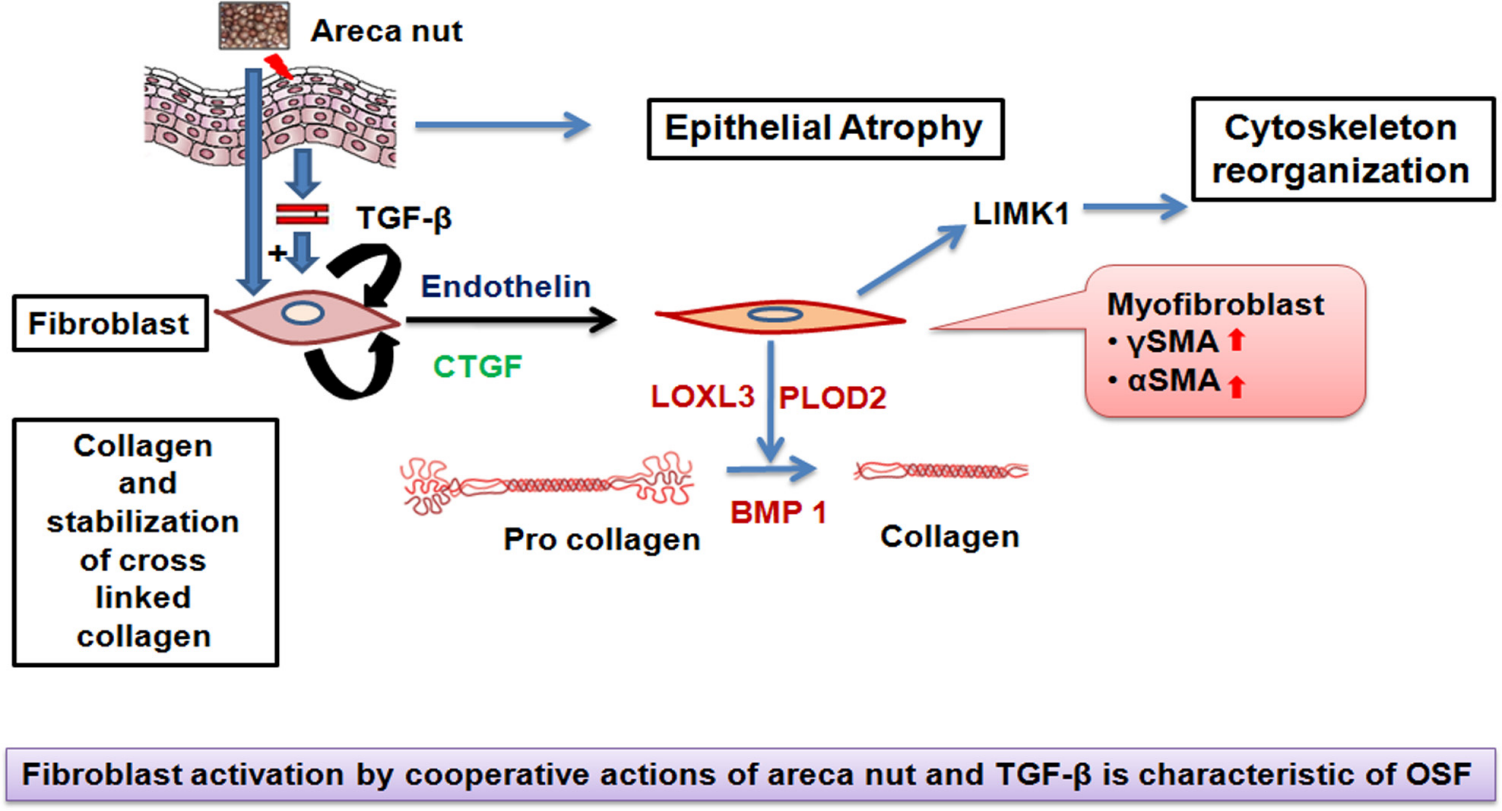
A model on the role of areca nut and TGF-β in OSF progression. Areca nut can induce and activate TGF-β in epithelial cells which can act together on the fibroblast cells and induce expression of other pro-fibrotic cytokines (Endothelin and CTGF). These cytokines can further enhance the fibrotic response and aid in conversion of fibroblasts to myofibroblasts expressing γSMA and αSMA markers. Areca nut and TGF-β can influence expression of cytoskeletal reorganizing protein LIMK1. The overall collagen production shall also increase. Collagen maturation and stabilizing enzymes (BMP1 and PLOD2 respectively) can also be induced by areca nut along with TGF-β. All these changes may lead to excessive deposition of extracellular matrix characteristic of OSF.

This study provides a comprehensive over view of fibroblast response to areca nut and TGF-β. We propose an important role of epithelium in OSF progression. Areca nut insult to the epithelium may injure the epithelium as well as induce pro-fibrotic factors; primarily TGF-β which along with areca nut alters the fibroblast phenotype by activation of a fibrogenic gene expression profile (Fig 11).
